# Myokines and Microbiota: New Perspectives in the Endocrine Muscle–Gut Axis

**DOI:** 10.3390/nu16234032

**Published:** 2024-11-25

**Authors:** Federica Saponaro, Andrea Bertolini, Riccardo Baragatti, Leonardo Galfo, Grazia Chiellini, Alessandro Saba, Giuseppina D’Urso

**Affiliations:** Department of Surgical, Medical and Molecular Pathology and Critical Care Medicine, University of Pisa, Via Roma 56, 56126 Pisa, Italy; andrea.bertolini@med.unipi.it (A.B.); r.baragatti1@studenti.unipi.it (R.B.); l.galfo@studenti.unipi.it (L.G.); grazia.chiellini@unipt.it (G.C.); alessandro.saba@unipi.it (A.S.)

**Keywords:** skeletal muscle, myokines, exerkines, gut microbiota, gut–muscle axis

## Abstract

This review explores the dual role of skeletal muscle as both a mechanical and endocrine organ, highlighting its contributions to overall health and its adaptability to various inputs such as nutrition, hormones, exercise, and injuries. In addition to its role in metabolism and energy conversion, skeletal muscle secretes signalling molecules called myokines (at rest) and exerkines (during/after physical exercise), which communicate with other organs like the brain, the cardiovascular system, and the immune system. Key molecules such as interleukins, irisin, and myostatin are discussed for their roles in mediating muscle health and inter-organ communication. This work also focuses on the muscle–gut axis, emphasising the bidirectional interaction between skeletal muscle and the gut microbiota, a complex ecosystem influencing immune defence, digestion, and metabolism. Muscle activity, particularly exercise, alters the gut microbial composition, promoting beneficial species, while gut-derived metabolites like short-chain fatty acids (SCFAs) impact muscle metabolism, mitochondrial function, and insulin sensitivity. Dysbiosis, or an imbalanced microbiota, can lead to muscle atrophy, inflammation, and metabolic dysfunction. This evidence highlights emerging research into myokines and exerkines as potential therapeutic targets for managing conditions like muscle decline, ageing, and metabolic diseases through muscle–gut interactions.

## 1. Introduction

Skeletal muscle constitutes 40% of the total body weight and is a unique organ due to its biochemical and metabolic functions [1]. It receives inputs from various sources, such as nutrition, hormonal balance, physical activity/exercise, and injuries, responding with a remarkable capacity for plasticity, remodelling, and the production of effectors, which, in turn, influence the overall body’s health [2]. From a mechanical perspective, skeletal muscle can convert chemical energy into mechanical energy, a capacity that directly depends on certain key morphological parameters [3]. Metabolically, skeletal muscle plays several roles, including contributing to basal energy metabolism, serving as storage for important substrates, maintaining core temperature, and consuming the majority of the oxygen and fuel used during physical activity and exercise [4].

From a biochemical point of view, skeletal muscle is now recognised as capable of establishing connections with other organs and tissues through its “secretome” [5]. However, the concept of skeletal muscle’s multiple integrated motor, endocrine, and neuromodulatory functions is a relatively recent intuition. Only in the early 2000s was skeletal muscle recognised as a producer of a variety of secretory moieties during resting time, namely, “myokines” and molecules termed exerkines, which are secreted after the stimulus of exercise [6]. Considering the skeletal muscle mass, it can be defined as the largest endocrine organ of the body [7]. The muscle secretome seems to be very diversified, including peptides, metabolites, and non-codifying small RNAs, such as microRNAs and circular RNAs (miRNAs and circRNAs, respectively), recently emerging as post-transcriptional modulators. Muscle exerkines and myokines are responsible for the intense crosstalk with other organs and systems, particularly the cardiovascular system, brain, kidney, pancreas, bone, immune system, and gut, which is the focus of this review and a subject of great interest in the recent literature [6].

The human gastrointestinal tract harbours a complex, dynamic, and living population of microorganisms, collectively known as the gut microbiota, which has been extensively studied and is known to have a profound influence on the host during health and disease [8]. This population is represented by bacteria, archaea, fungi, and viruses, numbering over one hundred billion, all living in a state of eubiosis with the human organism, providing mutual benefits. The composition of the gut microbiota can be altered by various factors, including diet, age, lifestyle, pregnancy, and diseases [9]. As a matter of fact, these organisms are known to be beneficial to our body, intervening in several processes, including important metabolic functions and protective mechanisms, and affecting the activities of various organs, including the brain, adipose tissue, endocrine pancreas, and skeletal muscle [8].

The aim of this review is to explore and revise the latest literature regarding the interesting and fascinating mutual and bidirectional interaction of endocrine muscle and gut microbiota, which may lead to new insights and therapeutic targets.

## 2. The “Endocrine” Muscle

As previously mentioned, the story of skeletal muscle as an endocrine organ capable of communicating with other organs through the secretion of various molecules has been recently written. More than 50 years ago, it was suggested that, during contraction, skeletal muscle cells could release a factor impacting metabolic processes, but this idea was mostly ignored for years [10].

In the early 2000s, researchers made a significant breakthrough regarding the benefits of physical activity, discovering that myocytes can release interleukin 6 (IL-6) during exercise, resulting in a rise in hepatic glucose production to fuel the muscles [11]. The term “myokine” was coined to describe cytokines or peptides produced by skeletal muscle cells and released into the circulation to influence other cells, tissues, or organs. IL-6 was identified as the first myokine, sparking increased interest in the muscle secretome or ‘myokinome’ [11].

The term “exerkines” is even more recent, referring to metabolites released in response to physical exercise, both acute and chronic. These substances include not only myokines themselves but also cardiokines, hepatokines, adipokines, baptokines, and neurokines, all of which contribute to overall health and homeostasis [12]. The intricate metabolic network of skeletal muscle, facilitated by the secretion of myokines and exerkines, positions this tissue as a key regulator of systemic metabolism [12,13]. In addition to myokines and other soluble factors, skeletal muscle-derived extracellular vesicles (EVs), including exosomes, have emerged as significant mediators of inter-organ communication [14]. These EVs, released from muscle in response to physical activity, carry a range of bioactive molecules—such as proteins, miRNAs, and metabolites—that function as signalling entities to distant organs, including the liver, adipose tissue, and brain. By facilitating the transport of these molecules, muscle-derived EVs enable skeletal muscle to regulate systemic metabolic processes and actively participate in whole-body homeostasis [14,15]. This exosome-mediated transport underscores the endocrine role of skeletal muscle beyond conventional myokines and exerkines, further establishing it as a critical regulator in inter-organ communication and metabolic health. However, research on myokines and exerkines has escalated in recent years, with numerous papers exploring this field and the “ever-expanding” secretome. In this review, we will focus on well-characterized molecules known to be involved in the muscle–gut axis crosstalk. Based on their chemical nature, they can be separated into protein-based (such as interleukins, irisin, and myostatin) and non-protein-based (for example, lactate, β-aminoisobutyric acid, and miRNAs).

### 2.1. Title Protein and Non-Protein-Based Myokines/Exerkines

#### 2.1.1. Protein-Based Myokines/Exerkines: Interleukins

In the overview of myokines, we start with the oldest. As already mentioned, IL-6 is one of the most studied, identified in 2000 [16]. Human IL-6 consists of 212 amino acids forming a four-helix bundle structure with four α-helices folded in a well-defined configuration. While elevated plasma levels of IL-6 are associated with negative effects, like insulin resistance and impaired glucose metabolism in cases of obesity and diabetes, its release following muscle contraction has been shown to exert local beneficial effects within the muscle [17,18,19]. This release is associated with an increased glucose uptake and fat oxidation through the activation of adenosine monophosphate (AMP)-activated protein kinase (AMPK) and/or phosphatidylinositol 3-kinase (PI3K). These effects are mediated by the binding of IL-6 to its specific transmembrane alpha receptor (ILRα), leading to the formation of a complex that triggers the homodimerisation of the glycoprotein (gp)-130 (also known as IL6Rβ), thereby initiating downstream signalling pathways [17].

Additionally, IL-6 may also exert distal effects. In the liver, it stimulates hepatic glucose production during exercise, while, in adipose tissue, it promotes lipolysis and facilitates the release of free fatty acids (FFA). These beneficial effects of IL-6 underscore the interplay between skeletal muscle, liver and adipose tissue. IL-6 released in response to exercise has also been associated with enhanced insulin secretion by stimulating glucagon-like peptide (GLP)-1 secretion from intestinal L-cells and pancreatic alpha-cells [20,21].

Interleukin-7 (IL-7) is also considered a myokine, although it is not produced by skeletal muscle. It has mainly been attributed to stromal cells in lymphoid organs and some non-lymphoid tissues, including the liver, skin, lungs and intestine. Its expression increases during muscle contraction and plays a relevant role in satellite cell differentiation [19]. A study on human myotube cultures derived from satellite cells confirmed the presence of IL-7, identified through immunoblotting and real-time PCR. IL-7 expression increased at both the mRNA and protein levels during cell differentiation [22].

Interleukin-15 (IL-15) is recognised as a T cell growth factor that accumulates in muscle after physical exercise, like other myokines, promoting myosin heavy chain (MHC) accumulation. Its overexpression supports myoblast differentiation and muscle mass growth, playing a significant role in skeletal muscle and adipose tissue interaction [18]. A study on young, healthy male volunteers engaged in regular physical activity found that, following endurance exercise, IL-15 mRNA levels increased in the triceps brachii, vastus lateralis, and soleus muscles, as observed through muscle biopsies. However, this increase in IL-15 mRNA did not correspond to an increase in IL-15 protein in muscle tissue or plasma. This suggests that an elevated level of IL-15 mRNA in skeletal muscle does not lead to protein production, indicating the existence of a translationally inactive pool of IL-15 in muscle [23].

#### 2.1.2. Myostatin

Myostatin, also known as growth differentiation factor 8 (GDF-8), belongs to the transforming growth factor beta superfamily (TGF-β). It is highly expressed in skeletal and cardiac muscle tissue, as well as in fat tissue [18]. Myostatin is synthesised as a precursor protein, undergoes proteolytic processing and cleavage of the signal sequence, and then expresses its biological activity circulating in the bloodstream and acting as a negative regulator of muscle mass growth [18,24]. Upon binding to its receptor, myostatin mediates its function through the receptor-associated small mother against decapentaplegic (Smad) proteins, Smad2 and Smad3, which form a heterodimeric complex with Smad4. The Smad complex is considered the intracellular mediator of myostatin pathways, translocating into the nucleus and activating the transcription of specific genes [25]. Myoblast proliferation is controlled by myostatin, which increases cyclin-dependent kinase inhibitor 1 (Cdk1-p21) expression and inhibits Cdk activity, leading to hypophosphorylation of retinoblastoma (Rb) and the arrest of the myoblast cell cycle. Upon receiving a differentiation signal, myoblast determination protein 1 (MyoD) is activated, triggering the expression of downstream myogenic genes, such as myogenin and p21, promoting the formation of committed myoblasts that fuse to form multinucleated myotubes [26]. Myostatin modulates this process by inhibiting MyoD expression via Smad3 after the differentiation signal, thereby suppressing downstream myogenic gene expression and myogenic differentiation [26]. Moreover, Smad3 expression alone is enough to enhance atrogin-1 promoter activity, suppress Protein kinase B (Akt)/mTOR (mammalian/mechanistic target of rapamycin) signalling and protein synthesis, and trigger muscle fibre atrophy [27]. Ultimately, myostatin acts as a potent inhibitor of muscle growth and regeneration, promoting muscle fibre atrophy and limiting muscle mass by suppressing myogenic differentiation and protein synthesis pathways.

In vivo studies show that skeletal muscle mass is significantly greater in myostatin-knockout mice than in wild-type mice. Similarly, humans with homozygous mutations in the myostatin gene exhibit unusually high muscle mass development. While the loss of myostatin function leads to increased skeletal muscle mass, it also has drawbacks. In myostatin-knockout mouse models, muscle growth is accompanied by reduced muscle strength, a higher proportion of fast-twitch fibres, and decreased mitochondrial density, indicating that muscle size gains may come at the expense of muscle function and endurance [24]. Myostatin is known to contribute to muscle mass reduction in older adults, whereas resistance exercise can slow down this process by decreasing myostatin blood levels and activating the Notch pathway in rat models [28].

#### 2.1.3. Irisin

Skeletal muscle and adipose tissue present a peculiar glycosylated type I cell membrane protein, FNDC5 (fibronectin type III domain-containing protein 5), which undergoes proteolytic cleavage of its extracellular tract during physical exercise and then is released into the peripheral circulation as irisin [29,30]. The primary action of irisin is related to adipocyte metabolism, permitting the browning of white adipose cells and thermogenesis. However, recent evidence suggests several protective roles of irisin on metabolically active organs and on the brain.

Irisin has been shown to have effects on the pancreas, where it enhances insulin secretion from pancreatic β cells. This can contribute to better regulation of blood glucose levels, which is critical for maintaining metabolic homeostasis [31]. By improving insulin sensitivity, irisin can help in better utilisation of glucose by tissues, thereby reducing blood sugar levels and lowering the risk of insulin resistance and type 2 diabetes. Some studies suggest that irisin may promote the health and function of pancreatic β cells, potentially protecting them from stress and apoptosis (cell death). This could help in preserving insulin production in situations of metabolic stress [32]. Irisin may also exert anti-inflammatory effects that could benefit the pancreas, especially in conditions like obesity and type 2 diabetes, where inflammation plays a significant role in pancreatic dysfunction. Increased levels of irisin have also been associated with neuroprotection. In fact, overexpression of the *FNDC5* gene or elevated irisin levels have been linked to reduced deposition of β amyloid peptides (Aβ40 and Aβ42), which are observed in the early stages of Alzheimer’s disease [29]. Overall, the effects of irisin on the pancreas suggest it plays a beneficial role in glucose metabolism and pancreatic health, but more research is needed to fully elucidate its mechanisms and therapeutic potential [30].

Clinical studies have shown that exercise in young adult athletes increases irisin plasma levels, which improves cognitive performance, while, in older adults, it helps reduce visceral adipose tissue [33].

Recently, in vitro and in vivo studies demonstrated further correlation of irisin with bone metabolism, as irisin promotes osteoblast proliferation, directly influencing bone density and geometry [29]. Moreover, irisin plays an autocrine role on the skeletal muscle itself: in vivo studies have demonstrated that knockout mice for irisin display a sarcopenic phenotype, which is rescued after irisin administration [34]. Indeed, cardiac muscle also seems to be an important source of irisin, as the heart is positively influenced by this myokine [35].

#### 2.1.4. Minor Protein-Based Myokines/Exerkines

Decorin is a small proteoglycan belonging to the SLRP (small leucin-rich proteoglycan) family, involved in several metabolic pathways, proliferation, and differentiation. It also binds to Toll-like receptors 2 (TLR2) and has the ability to trigger M1 macrophage differentiation [36]. Like previous exerkines, decorin is secreted into the circulation during muscle contraction and plays a role in muscle growth. It also binds to myostatin in a zinc-dependent manner, inhibiting its action, and may, therefore, be considered for its myogenic effects as a hypothetical target in muscle-wasting treatment [18]. In mouse models, overexpressing decorin in skeletal muscle promotes the expression of the myogenic factor Mighty, which is generally inhibited by myostatin, although Mighty is expressed across multiple tissues. Additionally, in C2C12 cells, overexpression of Mighty drives myoblasts to exit the cell cycle earlier, enhances differentiation, and promotes muscle hypertrophy. Mighty also stimulates higher expression of MyoD and IGF-II, further supporting muscle growth and development [37].

Apelins were initially identified as adipokines and participate in different processes, including cardiac functionality. They have been emerging as novel myokines with interesting roles. Vinel et al. recently identified apelin, a from 13 to 36 amino acid peptide, as an exercise-induced myokine that enhances muscle metabolism and stem cell function during ageing. Physical exercise has highlighted the crucial role of muscle cell progenitors, such as satellite cells, in muscle plasticity, showing that exercise promotes muscle regeneration by activating these stem cells. However, this regenerative capacity is reduced in older adults. Additionally, mitochondrial homeostasis may be essential in the functional decline of these stem cells, mirroring the effects observed in myofibers [38]. Its production declines with age in both humans and rodents, correlating positively with the benefits of exercise in older adults [38,39,40]. Mice lacking apelin or its receptor (APLNR) exhibited significant muscle dysfunction as they aged. Restoring apelin signalling improved muscle function through mechanisms such as mitochondrial biogenesis, autophagy, and anti-inflammatory responses, as well as enhanced regeneration via muscle stem cells [38]. Their findings establish a positive feedback loop between physical activity, apelin, and muscle function, suggesting that apelin could serve as an early diagnostic marker for sarcopenia. Modern techniques such as liquid chromatography coupled to mass spectrometry (LC-MS) have been demonstrated to be a gold standard for apelin measurements in the bloodstream [41].

Insulin-like growth factor 1 (IGF-1) regulates skeletal muscle growth and regeneration, and its normal expression is important to maintain the activity of signalling pathways like PI3K/Akt/mTOR and PI3K/Akt/glycogen synthase kinase-3 beta (GSK3β) [42]. By observing the phenotype of IGF-1 knockout mice, we understand how crucial this is for skeletal muscle as well as bone [42]. Adiponectin is an adipokine associated with anti-inflammatory and insulin-like effects that is secreted by adipocytes. Acute exercise, like running, raises plasma levels of adiponectin, although it decreases levels in cerebrospinal fluid [6,43]. For instance, in a study of young participants, serum adiponectin levels were measured before exercise and one hour after a 90 min run using protein arrays, ELISA, and immunoblotting techniques. Post-exercise, adiponectin levels in cerebrospinal fluid decreased by 21.3% (as measured by arrays) and 25.8% (by ELISA), with immunoblotting confirming a reduction specifically in low-molecular-weight adiponectin [43]. Heat shock protein 72 (HSP72) is a protein that is expressed at low levels under basal conditions but increases significantly in response to stress. Studies on adults have shown that acute exercise increases HSP72 expression, particularly when muscle glycogen levels are low. Muscle biopsies reveal that, in glycogen-depleted muscles, HSP70 gene expression remains unchanged under resting conditions but significantly increases following exercise. In contrast, muscles with normal glycogen levels do not exhibit this increase in HSP70 expression after exercise [44].

Other protein exerkines, such as secreted protein acidic and rich in cysteine (SPARC), are relevant in the cardiometabolic system, exerting paracrine and autocrine effects on muscle, tissue remodelling, and cell regulation. Syndecan 4 (SDC4) is involved in cell–extracellular matrix crosstalk, inflammation, and skeletal muscle growth [6].

### 2.2. Non-Protein-Based Myokines/Exerkines

Lactate has been traditionally viewed as a simple metabolic byproduct, but it is now recognised as involved in processes such as learning and memory. High-intensity interval training (HIIT) increases lactate blood levels and activates extracellular signal-regulated kinase (ERK) and Akt pathways. Like apelin, lactate exerts some of its effects through G protein-coupled receptors (GPCRs) [41]. Recent studies have also linked lactate accumulation in plasma to specific exerkine responses to training [45].

β-aminoisobutyric acid (BAIBA), derived from thymine catabolism, is now considered as a non-protein-based exerkine as well. BAIBA exerts beneficial effects on muscle mass, increasing mitochondrial FFA oxidation and protecting against inflammation mechanisms. It also induces the browning of white adipose tissue (WAT), similar to other myokines [46]. Another non-protein-based compound that rises in the bloodstream after exercise—and is primarily synthesised in the liver—is the ketone body β-hydroxybutyrate. This molecule binds to HCAR2/HCA2 receptors, triggering cytoprotective, anti-inflammatory signalling [47]. Additionally, β-hydroxybutyrate positively influences the microbiome, which may contribute to the protective effects of a ketogenic diet on epilepsy and other disorders [48].

The compound 12,13-dihydroxy-9Z-octadecenoid acid (12,13-diHOME) is a non-protein-based lipokine acting as a ligand for the peroxisome proliferator-activated receptor γ (PPAR-γ). This compound, derived from linoleic fatty acid metabolism, plays a significant role in glucose and adipose pathways. Nevertheless, it is also related to regulating systolic blood pressure [49]. Considered a baptokine because it is secreted by brown and beige adipose tissue, 12,13-diHOME affects not only adipose tissue but also skeletal and cardiac muscles. Its levels can increase in response to low temperatures and acute physical exercise [50].

Additionally, some miRNAs have shown potentially positive effects on cardiac muscle, such as miR-1, miR-17-3p, and miR-214 [12]. In sarcopenic models, it has been seen that specific genes such as *PTEN* produce miRNAs, like miR-222, that are involved in cell growth and proliferation processes. However, its altered expression can lead to gastric cancer or obesity [51].

In sarcopenia, changes in miRNA expression directly or indirectly affect key signalling pathways, including IGF-1, AMPK, mTOR, and PTEN. For example, miR-1, uniquely expressed in sarcopenia models, inhibits IGF-1, reducing miR-1 expression by 50%, and has been shown to increase muscle hypertrophy, confirming its role in this pathway. Similarly, miR-222 inhibits PTEN [51].

Another interesting miRNA is certainly miR-29 since its expression downregulates *CLDN1* and *NKRF,* leading to increased intestinal permeability, as observed in studies on knockout mice and in tissue samples from patients with irritable bowel syndrome (IBS) [52]. Certain miR-29 variants, such as miR-29a-3p and miR-29b-3p, are associated with both sarcopenia and frailty, and their overexpression is also observed in specific conditions, including type 2 diabetes. In skeletal muscle, this elevated expression contributes to insulin resistance, altered substrate oxidation, and impaired glucose homeostasis. Additionally, miR-29 variants are linked to pro-fibrotic states, with their expression inhibited by pathways like TGF-β and the Smad family. This inhibition reduces fibrosis and promotes collagen extracellular matrix (ECM) production. Notably, miR-29b correlates with appendicular skeletal muscle mass, suggesting that miR-29 variants may serve as a molecular link between sarcopenia and frailty mechanisms [51]. Studies on mice by Wang et al. demonstrated increased plasma levels of 12 different miRNAs after swimming exercise, with miR-1192 showing particular promise for its protective function against myocardial infarction [12].

CircRNAs, initially identified in viruses, are involved in both health and disease by regulating nuclear gene expression, influencing alternative splicing, and acting as “molecular sponges” to inhibit miRNAs, thereby reducing mRNA degradation [53]. In pancreatic cancer-related cachexia, the Zinc Transporter 4 (ZIP-4) activates cAMP response element-binding protein (CREB), a zinc-dependent transcription factor, and enhances miR-373 activity, which promotes muscle atrophy. Research by Xiuhui Shi et al. reveals that CircANAPC7, a circRNA, can counteract this effect by sponging miR-373, regulating CREB expression, and potentially preventing cancer-induced muscle wasting. This suggests circRNAs could have therapeutic potential against pancreatic cancer. Despite advances, much remains unknown about circRNAs, particularly their roles in cardiac hypertrophy [54].

## 3. Gut Microbiota

The human gastrointestinal tract harbors a complex and dynamic community of microorganisms, known as the gut microbiota. The gut microbiota consists of more than 10^14^ living microorganisms, including bacteria, fungi, archaea and viruses, co-existing with the human body either in a state of eubiosis (balance) or dysbiosis (imbalance) [55]. Advances in molecular biology techniques, particularly the sequencing of the 16S subunit of ribosomal RNA (16S rRNA), have allowed researchers to identify and classify the constituents of the gut microbiota. It is mainly constituted of anaerobic bacteria, both facultative and obligate, including *Lactobacilli*, *Streptococci*, *Enterococci*, *Enterobacteriaceae*, *Bifidobacteria,* and *Clostridia*. Generally, the gut microbiota of an adult individual is composed of six bacterial phyla: *Firmicutes*, *Bacteroides*, *Actinobacteria* (*Bifidobacteria*), and *Proteobacteria* (*Enterobacteriaceae*, *Escherichia coli*) account for nearly 94%, and there are small amounts of *Fusobacteria* and *Akkermansia muciniphila*. The most studied fungi in the gut microbiota include *Candida*, *Saccharomyces*, *Malassezia,* and *Cladosporium* [8,55].

The distribution of this microflora in the intestine is uneven due to chemical and nutritional gradients and immune activity. The microbial population in the small intestine, mainly in the duodenum, is limited due to higher acidity (bile acids) compared to other intestinal districts, high levels of oxygen, and the presence of antimicrobials. As a result, it is primarily composed of fast-growing facultative anaerobic species, *Lactobacillaceae* and *Enterobacteriaceae*. The highest microbial density is found in the colon, with the prevalence of fermentative anaerobic species, such as *Bacteroides*, *Bifidobacterium*, *Prevotellaceae*, *Rikenellaceae,* and *Clostridium*, which degrade non-metabolised polysaccharides from the small intestine. This composition is favoured by the great availability of nutrients, lower antimicrobial concentrations, and slower intestinal transit times [56,57].

The colonisation of the gastrointestinal tract by the microbiota is a complex process that begins before birth. Emerging evidence suggests that the microbiota may even be present in womb tissues, such as the placenta [55,58]. 16S rRNA-based sequencing studies have shown that early meconium is rich in genera such as *Leuconostoc*, *Escherichia-Shigella*, *Enterococcus*, *Streptococcus*, and *Lactococcus* [57]. At birth, the mode of delivery also appears to affect microbiota composition. Infants born through natural birth have an identical microbial community to the mother’s genital tract, which is rich in *Lactobacilli*, *Prevotella,* and *Atopobium*. In contrast, infants delivered by C-section harbour microbes from the parent’s skin flora, such as *Staphylococcus*, *Corynebacterium*, and *Propionibacterium*, and are colonised by facultative anaerobes such as *Clostridium* [59,60]. Subsequently, breastfeeding and weaning shape the child’s intestinal microbiota. The intestinal microbiota of naturally breastfed infants is usually colonised by *Bifidobacterium*, which is present on the surface of the mother’s breast, while artificially breastfed babies have a lower bacterial density and a greater abundance of potentially pathogenic species such as *Clostridiaceae*. By the age of one, a child’s gut microbiota features a characteristic abundance of *Akkermansia muciniphila*, *Bacteroides*, *Veillonella*, and *Clostridium* [59,61]. The composition of the intestinal microbiota is completed and stabilised in the adult, although it is always influenced by factors such as nutrition, lifestyle, physical exercise, diseases, drugs, antibiotics, and advancing age. In individuals over the age of 65, the microbial population shifts, with an increased abundance of *Bacteroidetes* phyla and *Clostridium* [61].

**Figure 1:**
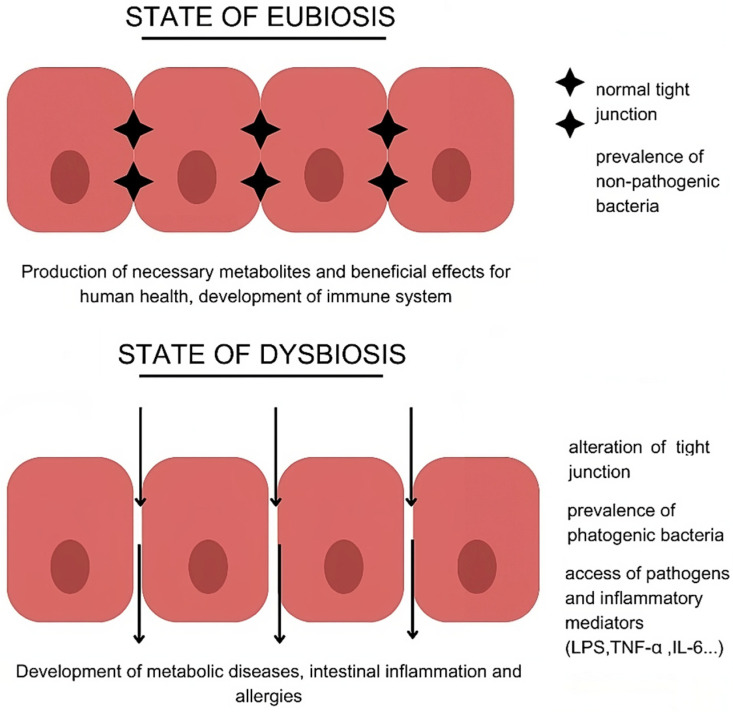
Representation of eubiosis and dysbiosis and their relative effects and consequences.

The intestinal microbiota can exist in two states: eubiosis and dysbiosis (Figure 1). Eubiosis refers to a state of microbial balance, in which the microbiota produces metabolites exerting beneficial effects for human health [8], such as those that support the immune system. Moreover, it provides a defensive line against potentially dangerous and pathogenic bacteria by competing for attachment sites on the brush border of enterocytes. It promotes proper intestinal function in the digestion and absorption of the diet, maintains the integrity of the intestinal barrier, and contributes to metabolic homeostasis. The microbiota plays a key role in producing vitamins (vitamin K, B vitamins, and folic acid), important metabolites for energy and glucose metabolism (short chain fatty acids (SCFAs) [62], synthesising amino acids and neurotransmitters, and regulating crucial connections, like the intestine–brain axis and the intestine–muscle axis [57]. Dysbiosis refers to an imbalance in the body’s microbial community, which can interfere with normal functions and potentially contribute to the onset of various diseases, including metabolic, inflammatory, intestinal, and allergic conditions. In dysbiosis, the stability of microbiota is disrupted, leading to reduced microbial diversity, a loss of beneficial bacteria, or an overgrowth of harmful ones. This imbalance can be triggered by factors specific to the host, such as genetics, lifestyle, infections, and inflammation, as well as external factors like diet, antibiotics, and hygiene. As a result, dysbiosis can impair bodily functions and lead to intestinal issues like IBS, inflammatory bowel disease, celiac disease, and colorectal cancer. It can also contribute to non-intestinal conditions, including metabolic disorders (e.g., obesity, type 2 diabetes) and central nervous system disorders like Alzheimer’s and Parkinson’s diseases [63].

## 4. Gut–Endocrine Muscle Axis: The Effects of Skeletal Muscle on Microbiota

There is substantial published evidence regarding the communication between skeletal muscle and the gut, suggesting the existence of a true axis between the two. The gut–endocrine–muscle axis displays bidirectional communication, and virtuos (positive) or vitious (negative) circles can be established in healthy, exercised people or in patients with chronic diseases. In this axis, virtuous cycles can emerge in healthy individuals who exercise regularly: exercise improves gut health, which, in turn, supports better muscle function and overall health. Conversely, vicious cycles can occur in individuals with chronic diseases: poor muscle function or a sedentary lifestyle can negatively impact gut health, which then worsens muscle health, creating a reinforcing loop of decline. In this section, we attempted to dissect the available data regarding the effects of myokines on the gut and microbiota. IL-6, whose secretion increases in response to exercise, has been found to improve glucose tolerance by stimulating glucagon-like peptide 1 (GLP-1) secretion. Incretin hormone GLP-1 levels increase when nutrients reach the intestinal L cell; nonetheless, they also grow in response to physical exercise. This suggests how exercise, through the secretion of myokines, can influence L cells’ secretion [64].

Irisin was shown to positively affect the microbiota in cases of pathogen-induced dysbiosis, such as ulcerative colitis (UC). In fact, in a mouse model of UC, it has been demonstrated how the administration of exogenous irisin impacts the gut microbiota, exerting/as well as exerting anti-inflammatory effects. The latter ability is related to the inhibition of cytotoxicity and apoptosis mechanisms [65]. UC is known to be associated with gut microbiota imbalances and immune dysfunction, leading to intestinal damage. Huangfu et al. divided 40 six-week-old C57BL/6 mice (20 males and 20 females) into four groups (control, irisin, dextran sulfate sodium (DSS)-induced UC, and DSS + irisin, 5 males and 5 females for a total of 10 mice for each group) and found that irisin significantly reduced inflammation in UC mouse models, as indicated by improved histological and inflammatory markers. Analysis of gut bacteria showed that irisin altered microbial diversity, with specific bacteria such as *Alloprevotella* and *Lachnospiraceae-UCG-001* being negatively correlated with inflammation, while *Bacteroides* was positively correlated with it [66]. Liu Xing and his research team discovered that irisin-knockout mice displayed anxiety- and depression-like behaviors, alongside significant alterations in the diversity and richness of their gut microbiota. At the phylum level, these mice showed a reduction in *Firmicutes* and an increase in *Bacteroidota*. At the genus level, they had notably lower levels of *Lactobacillus* and *Bifidobacterium*. Additionally, the absence of the irisin gene led to a significant decrease in N-desmethyl-mifepristone (RU 42633) and an increase in (-)-stercobilin levels. Analysis using the KEGG database revealed that the irisin-affected microbial metabolites were primarily involved in arginine and proline metabolism and influenced the mTOR signaling pathway [67].

Luo et al. investigated the role of the myostatin (*MSTN*) gene in regulating intestinal structure, microbiota composition, and muscle growth. The researchers found that deleting the *MSTN* gene upregulates the expression of tight junction-related genes (*TJP1* and *OCLN*) via the myosin light-chain kinase pathway, leading to structural changes in the intestines of *MSTN*−/− pigs, such as a thicker muscularis and longer plicae. These alterations influenced gut microbiota composition, enriching SCFA-producing microbes. Transplantation of these microbiota into mice promoted larger myofibers and increased glycolytic muscle mass. Elevated SCFA levels, particularly valeric acid, activated the Akt/mTOR pathway, promoting type IIb myofiber growth and preventing dexamethasone-induced muscle atrophy [68].

Research showed that apelin is a key regulatory peptide in the gastrointestinal system with cytoprotective roles that also influence food intake and hydration. In young rats, apelin-13 impacted cellular processes like apoptosis and mitosis in various gut regions, affecting tissue maturation and altering DNA repair enzyme (OGG 1/2) expression, with effects differing by administration route [69].

Apelin also supported metabolic regulation through the gut–brain axis, which controls glucose homeostasis. Fournel et al. found that apelin modulates intestinal contractions via the enteric nervous system, enhancing glucose uptake in muscle through hypothalamic nitric oxide (NO) release—a promising pathway for treating obesity and diabetes [70].

Additionally, apelin may help reduce gut barrier dysfunction and visceral hypersensitivity, key issues in IBS. Nozu et al. found that apelin modulates signaling pathways linked to IBS inflammation, with apelinergic signaling blockade decreasing hypersensitivity and colonic permeability. These findings suggest apelin as a promising target for managing IBS symptoms [71].

Finally, certain miRNAs, including miR-29, have demonstrated significant influence on gut health by contributing to gut barrier integrity through various mechanisms. Specifically, miR-29 plays a role in downregulating genes like *CLDN1* and *NKRF*, which can lead to increased intestinal permeability, a phenomenon observed in studies on knockout mice and tissue samples from IBS patients. Moreover, miR-29 variants, such as miR-29a-3p and miR-29b-3p, are linked not only to gut health but also to systemic conditions like sarcopenia, frailty, and type 2 diabetes, where elevated miR-29 expression affects insulin resistance, glucose homeostasis, and inflammation. Additionally, miR-29 is implicated in immune modulation and pro-fibrotic states, where its expression is inhibited by TGF-β and SMAD pathways, reducing fibrosis and enhancing collagen extracellular matrix (ECM) production. Through these diverse effects, miR-29 contributes to epithelial repair, immune balance, and interactions with gut microbiota, making it essential to both gut and systemic health [51,52].

Despite the small number of studies on model animals and humans, there is growing evidence of a muscle–gut axis involved in the pathophysiology of physical frailty and sarcopenia, although the causal link still remains uncertain. Whatever the case, it should be pointed out how physical activity has a positive effect on the modulation of the gut microbiota and how it acts by improving the biodiversity of taxa present in our gastrointestinal tract. This is, citing one more example, supported by a study by Estaki et al., in which butyrate levels and levels of butyrate-producing species (*Faecalibacterium prausnitzii*) were found to be increased in mice that performed voluntary physical activity on a running wheel [72]. Similarly, lean young adults showed improved microbiota composition, physical performance, and SCFA production compared to obese adults [73]. Moreover, running athletes seem to experience improvements in their microbiota, particularly in the *Veillonella* genus, after exercise [74]. Indeed, a sedentary lifestyle is often associated with dysbiosis, as well as with a supernumerary growth of opportunistic pathogens and a malfunctioning of the commensal intestinal flora [75]. According to a recent study, the commensal flora remodelling induced by physical activity appears to be independent of age and exercise type, even if there is a possibility that it may be less pronounced in older subjects [76]. Low-intensity continuous exercise has been shown to positively affect gut microbiota by promoting the growth of beneficial species such as *Bifidobacterium spp*., *R. hominis*, *A. muciniphila*, and *F. prausnitzii* [77]. Moderate training correlates with increased biodiversity of species inhabiting the gastrointestinal tract by increasing the expression of genes involved in protein and carbohydrate metabolism as well as SCFA production [78,79]. While exercise generally improves gut health, overtraining in professional athletes can affect the normal balance of the gut microbiota, leading to intestinal ischemia and oxidative stress. Moreover, this promotes the proliferation of pathogens and related toxins, leading to states of local or systemic inflammation, due to increased permeability of intestinal blood vessels [80]. So, upon careful inspection, it becomes clear how the gut microbiota is interconnected with metabolic and inflammatory states and with muscle function.

## 5. Gut–Endocrine Muscle Axis: The Effects of Microbiota on Skeletal Muscle

**Figure 2:**
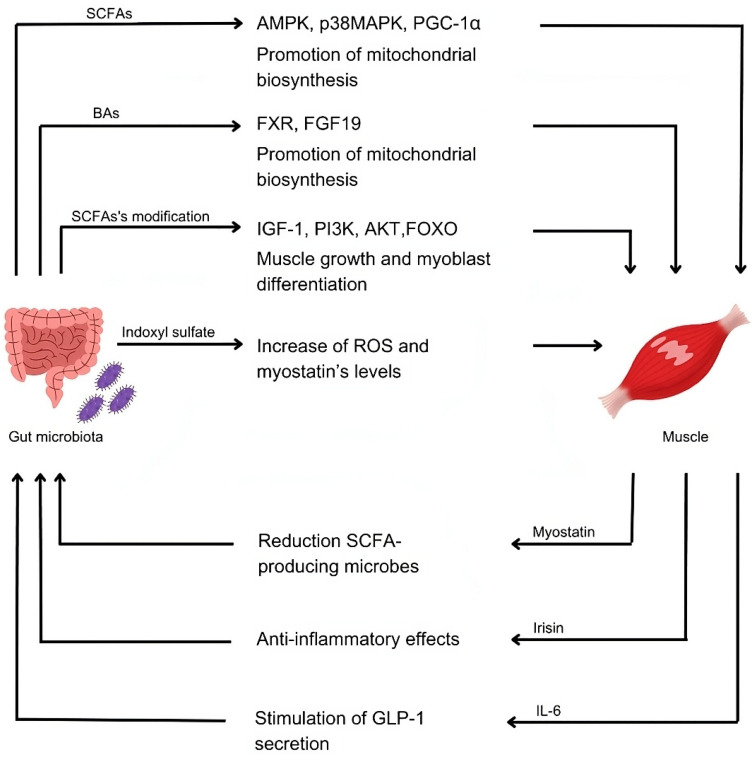
Summary of the major components involved in the muscle–gut axis.

Recent studies have demonstrated that the gut–muscle axis is bidirectional, with the quality and quantity of intestinal microbiota influencing muscle function and metabolism (Figure 2). The gut microbiota can impact muscle mass and function by regulating energy metabolism, endocrine and insulin sensitivity, immune and inflammatory responses, protein synthesis, and myokine production [77]. Additionally, the gut microbiota can modulate the duration and quality of physical exercise and host fitness and serves as a potential biological target for preventing and treating muscle-related diseases such as sarcopenia and muscular dystrophy [77].

In vivo studies on mice and piglets have shown that germ-free (GF) animals, which lack intestinal microbiota, exhibit muscle atrophy and reduced skeletal muscle weight compared to conventional, pathogen-free (PF) animals with intact microbiota and immune systems [81,82]. GF animals also display reduced mitochondrial DNA content, signalling impaired mitochondrial biogenesis and growth. This phenotype was rescued by faecal transplantation from PF animals to GF animals, resulting in the complete restoration of muscle mass and mitochondrial DNA content. Other studies have found that animals treated extensively with antibiotics, which disrupt the gut microbiota, experience muscle atrophy, reduced muscle weight, decreased running endurance, and increased muscle fatigue. Reintroducing adequate microbial flora to these animals increased their muscle mass and muscle-to-body weight ratio [77,81,82]. In GF mice, the absence of gut microbiota led to a greater loss of skeletal muscle mass, reduced IGF-1 levels, and decreased expression of genes involved in muscle growth compared to PF mice. This microbial deficiency also triggered increased expression of genes linked to muscle degradation, such as Forkhead box O3 (FoxO3), Atrogin-1, and muscle RING finger 1 (MuRF1), while downregulating MHC genes. Atrogin-1 and MuRF1, key muscle-specific ubiquitin ligases involved in muscle atrophy, promote protein breakdown via the ubiquitin-proteasome system, with MuRF1 targeting myofibrillar proteins and Atrogin-1 affecting protein synthesis [81,83].

The gut microbiota also influences skeletal muscle by modulating inflammation and immune function. A healthy gut microbiota helps maintain immune function by interacting with the intestinal barrier. Dysbiosis, or an imbalance in gut microbial homeostasis, can lead to an overgrowth of harmful microbes and a reduction in beneficial ones, which compromises the gut barrier [84]. This allows harmful substances, such as lipopolysaccharide (LPS), to enter the bloodstream. LPS, a potent endotoxin from the outer membrane of Gram-negative bacteria, activates Toll-like receptor 4 (TLR4) signalling. Upon binding to its ligand, TLR4, along with its co-receptors, interacts with adaptor proteins like Myeloid differentiation primary response 88 (Myd88), triggering downstream signalling pathways that activate IκB kinase–nuclear factor κB (NFκB), MAPK pathways, ERK, and c-Jun N-terminal kinase (JNK) [85]. This cascade promotes systemic inflammation through the secretion of proinflammatory cytokines, such as tumour necrosis factor-alpha (TNF-α) and IL-6, and impairs insulin receptor signalling, leading to reductions in muscle mass and function. Although these inflammatory pathways have been linked to the loss of skeletal muscle mass and function in frail elderly individuals, it remains unclear whether enhanced signalling through the TLR4–NFκB–MAPK network plays a role in age-related skeletal muscle insulin resistance in otherwise healthy older adults.

Ticinesi et al. examined how diet and physical activity impact gut microbiota and contribute to primary sarcopenia, a geriatric syndrome primarily caused by ageing but exacerbated by factors like poor diet, endocrine dysfunction, and inflammation [75]. A study by Ponziani F.R. et al. highlighted that, in patients with cirrhosis, sarcopenia was associated with elevated serum levels of interleukins, including IL-1 beta, IL-2, and IL-6. These inflammatory imbalances were linked to a reduced presence of bacteria commonly associated with physical activity (such as *Methanobrevibacter*, *Prevotella*, and *Akkermansia*) and an increased presence of *Eggerthella*, a gut microbial marker of frailty [86]. The study also noted a higher abundance of potentially pathogenic bacteria like *Klebsiella*, which came at the expense of beneficial autochthonous bacteria [86]. Using 16S rDNA sequencing and LC-MS-based metabolomics profiling, a recent study by Zhou J et al. analysed faecal samples from 30 sarcopenic and 30 non-sarcopenic older adults. They identified significant alterations in 29 bacterial genera and 172 metabolites, with *Blautia, Lachnospiraceae*, and *Subdoligranulum* showing potential for sarcopenia diagnosis. Correlation analysis revealed that IL-6 levels were negatively linked with *Blautia*, and metabolic pathways like purine, arginine, and histidine metabolism were implicated in sarcopenia development. This study supports the gut–muscle axis hypothesis, offering insights into pathogenic processes and potential treatments for sarcopenia [87].

The gut microbiota also regulates skeletal muscle metabolism by producing metabolites, such as SCFAs and secondary bile acids (BAs), which directly influence myocytes. SCFAs, primarily acetate, propionate, and butyrate, are produced by the fermentation of fibres, like non-digestible carbohydrates, by gut bacteria. A study by Li. G et al. has shown that SCFAs inhibit LPS-induced inflammation through GPCRs and the inhibition of histone deacetylases (HDACs) [88]. SCFAs are increasingly recognised as modulators of skeletal muscle metabolism via their action on the G protein-coupled receptors FFAR3 (formerly known as GRP41) and FFAR2 (formerly known as GRP43). Additionally, GLP-1 signalling through its receptor (GLP-1R) activates PI3K and increases glucose transporter type 4 (GLUT4) expression, thereby enhancing glucose uptake in muscle cells. GLP-1 also increases microvascular perfusion in muscle, further supporting its role in skeletal muscle function [89]. SCFAs contribute to metabolic function and insulin sensitivity primarily through the activation of the AMPK pathway. They induce AMPK phosphorylation in skeletal muscle, increasing AMP concentrations and the AMP/ATP ratio. Phosphorylated AMPK activates p38 mitogen-activated protein kinase (p38MAPK) and peroxisome proliferator-activated receptor gamma coactivator 1-alpha (PGC-1α), which promotes mitochondrial biogenesis and enhances the oxidative metabolism of fatty acids [90,91,92]. Studies in both animals and humans have highlighted the effects of SCFAs on muscle health. Inoculation with butyrate-producing bacteria, such as members of the *Lachnospiraceae* family, resulted in a significant reduction in age-related sarcopenia. Additionally, older adults with normal muscle mass exhibited higher levels of butyrate compared to those with low muscle mass, with butyrate promoting mitochondrial biogenesis. Some studies have also found that older individuals with low muscle strength, but higher SCFA levels, had greater muscle strength than those with lower SCFA levels, suggesting a potential role of SCFAs in enhancing muscle function [77]. A recent study demonstrated that a specific combination of SCFAs (acetate: propionate: butyrate in a 60:20:20 ratio) increased glucose uptake in murine C2C12 myotubes, providing sufficient energy for physical exercise [93].

Bile acids (BAs), another metabolite produced by gut microbiota, play a role in muscle metabolism as well. The intestinal microbiota deconjugates primary bile acids secreted by the liver into secondary bile acids, which escape enterohepatic circulation and enter systemic circulation [83]. These BAs bind to cellular receptors like the farnesoid X receptor (FXR), which modulates glucose and lipid metabolism. FXR activation in the ileum stimulates the production of fibroblast growth factor (FGF) 19, which promotes mitochondrial biogenesis and muscle fibre size through the AMPK/PGC-1α pathway [88,94]. Lastly, autophagy, an essential process for cellular homeostasis, is also regulated by AMPK and PGC-1α signalling. Activation of these pathways declines with age and dysbiosis, reducing autophagic and mitophagic activity and leading to the accumulation of dysfunctional mitochondria, further exacerbating inflammation and contributing to muscle loss [88,95].

However, gut microbial metabolites can also negatively affect muscle function. For example, indoxyl sulfate, a uremic toxin with proinflammatory properties, accelerates muscle atrophy by increasing reactive oxygen species (ROS) in myoblast cells, promoting myostatin mRNA expression, and inhibiting cell proliferation. Similarly, p-cresol sulfate promotes insulin resistance and increases lipid accumulation in muscle. These metabolites increase with age but decrease with exercise and improved muscle strength [88].

## 6. Conclusions

This review underscores the pivotal role of skeletal muscle as both a mechanical and endocrine organ intricately involved in maintaining overall body health through its interaction with various systems, including the gut microbiota. Skeletal muscle not only supports movement and metabolism by converting chemical energy into mechanical energy, but it also communicates with distant organs via the secretion of myokines and exerkines. These signalling molecules, such as IL-6, irisin, and myostatin, mediate metabolic processes, regulate inflammation, and influence muscle growth, contributing to systemic health. Emerging research on the gut–muscle axis reveals a bidirectional relationship, wherein exercise positively influences gut microbial diversity and composition, while the gut microbiota, in turn, impacts muscle function and metabolism. This interaction highlights the importance of maintaining gut microbial balance (eubiosis) to support muscle health and prevent dysfunctions like atrophy and inflammation. Furthermore, advancements in molecular biology have shed light on the complex and dynamic nature of the gut microbiota, emphasising its influence on immune defence, metabolic regulation, and communication with organs like the brain and skeletal muscle. This integrated understanding of muscle and gut health opens new avenues for therapeutic strategies targeting myokines, exerkines, and gut microbiota to address age-related muscle decline, including sarcopenia, metabolic diseases, and other health conditions. Ultimately, the interplay between skeletal muscle and the gut microbiota represents a critical axis in maintaining optimal health and preventing disease.

## Data Availability

The data provided in the current review are available from the corresponding authors on request.
